# Project STARLIT: protocol of a longitudinal study of habitual sleep trajectories, weight gain, and obesity risk behaviors in college students

**DOI:** 10.1186/s12889-019-7697-x

**Published:** 2019-12-23

**Authors:** Andrea T. Kozak, Scott M. Pickett, Nicole L. Jarrett, Shaunt A. Markarian, Kari I. Lahar, Jason E. Goldstick

**Affiliations:** 1 Department of Psychology, Oakland University, Rochester, MI USA; 2 Center for Translational Behavioral Science, Florida State University, Tallahassee, FL USA; 3 Department of Emergency Medicine, University of Michigan, Ann Arbor, MI USA; 4 Injury Prevention Center, University of Michigan, Ann Arbor, MI USA

**Keywords:** Obesity, Sleep, Diet, Physical activity, Body fat composition, Dual X-ray absorptiometry, Young adults

## Abstract

**Background:**

Obesity in the United States is a serious and preventable health concern. Previous research suggests that habitual short sleep may influence obesity-risk behaviors, such as increased caloric intake, decreased physical activity and increased engagement in sedentary activities (e.g., media consumption, computer usage). Given that existing longitudinal research studies have methodological concerns preventing conclusive interpretations, Project STARLIT was designed to address these limitations and identify future intervention targets.

**Methods:**

A sample of young adults (*n* = 300) will be recruited during the summer prior to entering college. Participants will be screened for eligibility requirements prior to the inclusion in the Time 1 assessment though phone and in-person interviews. Once enrolled, participants will complete four assessments over a two year period (i.e., approximately 8, 16 and 24 months after Time 1). Each assessment will consist of one week of data collection including both objective (i.e., habitual sleep, physical activity, body fat composition) and subjective (i.e., sleep diary, 24-h food recall, technology use, and sleep-related beliefs/behaviors) measures.

**Discussion:**

Project STARLIT is designed to address methodological concerns of previous research. In addition to clarifying the relationship between habitual short sleep and weight gain among young adults, the proposed study will identify problematic obesity risk behaviors associated with habitual short sleep (e.g., increased caloric intake, physical/sedentary activity). The results will identify prevention or intervention targets related to obesity risk.

**Trial registration:**

ClinicalTrials.gov NCT04100967, 9/23/19, Retrospectively registered.

## Background

Obesity is a serious problem among U.S. adults given its high prevalence, significant associated costs, associated poor health-related quality of life, and link to cardiovascular disease and cancer, which are the top two causes of death [1–5]. Obesity prevention through healthy diet and activity is important across the lifespan. However, individuals in college are prime intervention targets because evidence suggests that lifelong physical activity habits are often set during this time [6]. College students are a high-need group in terms of poor physical activity and dietary habits. According to a survey from the American College Health Association of over 19,500 undergraduate students from 40 U.S. community colleges and universities, only 42.6% of college students meet physical activity guidelines set forth by the American College of Sports Medicine and the American Heart Association [7]. A majority of these surveyed students also reported not following the U.S. government’s recommendation of consuming 5 or more fruits and vegetables each day [7].

Although unhealthy diet and activity choices are essential to explaining obesity, inadequate sleep may also play an important role as it is hypothesized to be directly and indirectly associated with obesity. Sleep is considered inadequate if one’s average nightly sleep duration is < 6 h per night, and is termed habitual short sleep. Almost 18% of U.S. adults are considered to be short sleepers [8]. The most recent American College Health Association survey found that 24% of the students reported sleep difficulties, 34.9% reported problems with daytime sleepiness on five or more days, and 14.4% reported that there were no days during the week in which they had obtained enough sleep to feel rested [7]. Over 64% of surveyed students expressed an interest in receiving sleep-related information from their university, but only 24% of surveyed students had actually received information. Further, there has been evidence for a trend among adults to obtain less sleep [9]; this problematic sleep trend appears to coincide with increased rates of excess weight over the past several decades [10].

Habitual short sleep in adulthood is associated with weight gain and obesity on the basis of body mass index BMI [11, 12];. Interestingly, some research has found this relationship to be stronger among children and adolescents [13], which suggests the importance of understanding this association in college students. Adults who sleep less than 5 h a night are 55% more likely to be obese in observational studies of habitual sleep [14]. Dual-energy x-ray absorptiometry (DXA) is a more accurate option than BMI for measuring body fat mass [15], but the relationship between shorter sleep and adiposity as determined through DXA is less clear than when using BMI. Some cross-sectional work supports a significant relationship between shorter sleep duration and higher body fat percentage among females [16–18]. However, body fat percent and short sleep were not related in males or females from one cross-sectional study [19]. On the other hand, a randomized crossover study found that males and females who were required to sleep only 5.5 h per night and were calorie restricted over the course of two weeks had a lower percent of body fat loss compared to when they slept 8.5 h and were calorie restricted [20].

An important component of the causal pathway between sleep and weight that is currently missing from the literature is how habitual sleep patterns correspond to patterns of energy expended through physical activity. Yet, it is known that short sleep is related to behavioral changes in energy intake. Specifically, shorter habitual sleep has been related to increased consumption of high calorie foods in 9–11 year olds [21] and increased total caloric intake and fat consumption in older women [22]. Further, there was no association between habitual sleep and energy intake in adolescents; however, sleeping more than 3 h during the day, which may be indicative of poor nighttime sleep, was associated with greater caloric intake [23]. Several experimental studies have found that increased caloric intake occurred after a series of nights, ranging from 2 to 14 days, of experimentally induced short sleep [ranging from 4 to 6 h per night [24–27]. Although a majority of these experimental studies have demonstrated a general increase in caloric intake, some studies have found an increase in calories from unhealthy foods, such as foods high in fat [24] and snack foods [26]. However, these experimental studies did not show a change in physical activity. One study found that two nights of short sleep (4.25 h) was linked to less vigorous physical activity after the first night and a decrease in total physical activity during the day [28]. We are not aware of any habitual sleep studies with adolescents that have included objective observations of physical activity. It may also be that the identification of characteristic sleep trajectories, rather than cross-sectional data or traditional modeling, would best represent the relationship between habitual short sleep and weight gain and obesity risk behaviors (i.e., high caloric intake, low physical activity).

This paper describes the protocol of Project STARLIT, which is a two-year prospective longitudinal study examining the relationship between habitual sleep and weight gain, along with obesity risk behaviors in a sample of Freshmen college students recruited during new student orientation. There appears to be a critical period in which habitual sleep changes have a maximum impact on weight gain [29]; therefore, recruiting recent high school graduates prior to the start of college may be an opportune time to observe the onset or exacerbation of habitual short sleep. The primary hypothesis is that we will observe different habitual sleep trajectories over time. The secondary hypothesis is that two sleep trajectories (stable habitual short sleep and increasingly shorter habitual sleep across time) will be significantly related to weight gain, increased body fat percent, and weight gain risk behaviors (i.e., increased average daily caloric intake and less daily average time spent engaging in moderate- and vigorous-intensity physical activities). We are not aware of any other studies that have attempted to relate sleep trajectories to behaviors most indicative of weight gain and obesity risk. This study will use objective measures of sleep, physical activity, and body fat composition as well as a gold standard measure of caloric intake; objective measurement is important because previous longitudinal investigations have primarily used self-report measures of sleep and physical activity, which can provide inaccurate information [23]. The supplemental hypothesis is that the two sleep trajectories (stable habitual short sleep and increasingly shorter habitual sleep across time) will be significantly associated with higher rates of media and technology use and higher rates of problematic sleep-related beliefs/behaviors (e.g., sleep difficulties, delayed sleep scheduling, sleep fragmentation, sleep hygiene problems, alcohol and caffeine usage, dysfunctional beliefs, napping).

## Methods/design

### Participants and eligibility criteria

This observational, longitudinal study will enroll a total of 300 undergraduate students. In order to be eligible for the study, individuals have to be first year college students in the traditional college age category of 18–22 and have a measured body mass index between 18.5 and 29.9. Exclusion criteria for the study include 1) pregnancy (due to DXA radiation exposure), 2) lacking the capability to be ambulatory (due to physical activity as a primary outcome), 3) currently taking a medication that could influence or interfere with sleep, or 4) reporting a past or current neurological problem, past or current head injury, past or current sleep disorder, current mental disorder (mood, anxiety, or substance use disorder), current psychosis, or current suicidal ideation or plans (due to the impact that these conditions may have on sleep, protocol adherence, or participant safety).

### Recruitment and screening process

#### Recruitment

Participants will be recruited during summer freshmen orientation sessions. All students will be provided with a card in which they will indicate whether or not they are interested in being contacted for future Psychology Department research study opportunities. The following information will be entered into a registry for students who want to be contacted: name, phone number, and email address. Participants will be randomly selected from the registry and provided information via phone, text, and/or email about the study purpose and requirements. They will also be informed about the eligibility criteria as required by the Humans Subjects Institutional Review Board (HSIRB) at the first author’s university. For example, they will be told they need to be ambulatory and this will be verified once they come into the laboratory. Interested students will be scheduled for an individual in-person session (Session 1) that involves two components: 1) screening (all potential participants), and 2) training (only eligible participants). If the sample size is not met during the first summer of recruitment, then a second cohort of students will be recruited using the same method the following summer.

#### In-Person screening (session 1)

After participants’ age is verified via photo ID, the informed consent process will be completed. Participants will read the consent form and a research assistant will answer any questions before participants sign two copies of the consent form (one for the participant and one for the researchers). Next, eligibility will be established on the basis of 1) a screening interview to assess for self-reported medication use and current or a history of a neurological problem, head injury, or sleep disorder, 2) the administration of the DSM-5 Self-Rated Level 1 Cross-Cutting Symptom Measure – Adult [30] and any relevant DSM-5 Self-Rated Level 2 measures [31] to assess for current mental disorders, 3) measurement of weight and height, and 4) a pregnancy test. Participants will provide the following information about all prescribed and over-the-counter medications as well as any herbal/nutritional supplements they are taking: name, dosage, reason for use, and amount of time they have been taking the medication or supplement. Participants will wear a hospital gown and no shoes to have their weight and height measured with a balance beam scale (Cardinal Detecto Digital Physician’s Scale, Model #6449) in order to calculate body mass index (BMI). Female participants will take a urine pregnancy test because of the radiation exposure from a DXA scan to be conducted after eight days of at-home data collection.

### Study procedures

#### Training (session 1)

Eligible participants will attend the training portion of the session in which they will learn portion size estimation using multiple methods [i.e., food/beverage models, color photographs of food and beverage items, and objects such as a baseball and deck of cards [32];. Participants will be taught how to record all food and beverages (including alcoholic beverages) in the National Cancer Institute’s Automated Self-Administered 24-h Dietary Assessment Tool [ASA24 [33, 34];. During this session, they will be asked to enter food and beverages consumed during the day and evening before the session. Research assistants will emphasize the importance of accurate entries (i.e., correct identification of a teaspoon vs. tablespoon, 1 oz vs. 2 oz, etc.) as well as not forgetting to enter any condiments. Next, participants will be instructed to wear a Phillips Respironics Actiwatch 2 device on the non-dominant arm and an Actigraph wGT3x-BT accelerometer device on the right hip; both devices were to be worn at all times except when bathing or swimming. Finally, participants will be trained on how to record in a daily diary the following information: sleep information, substance use, media and technology use, prescribed and over-the-counter medications and herbal/nutritional supplements taken, and time of day, duration, and for what purpose monitors were removed. Participants will be given copies of all training materials on a flash drive and on paper to facilitate ease of recording food/beverages and the wear/care of monitors and to encourage adherence.

#### Recording periods

During four separate periods over the course of two years, participants will complete eight consecutive days of at-home data collection. Participants will wear the Actigraph and Actiwatch devices 24 h a day except for when bathing or swimming during these recording periods. They will also complete the daily diary. Food and beverages will be recorded in the ASA24 at the end of the day. A Project Coordinator will review ASA24 data daily and will send an evening text reminder to participants who have yet to enter their food and beverages for the day. Food and beverages must be entered by midnight because the ASA24 does not allow for retrospective data entries. If several days of ASA24 data are not entered, then a Project Coordinator will send a follow-up text to discuss any difficulties the participant might be having with timely data entry. Prior to sessions 3–5, participants will attend a brief appointment to receive portion size estimation booster training; entering food and beverage data in the ASA24; wearing and caring for the devices; and how to record necessary sleep information in the daily diary. The participant’s username and password for the ASA24 will also be checked to make sure they still work.

#### Data collection sessions (sessions 2–5)

After each eight-day at-home data collection period, participants will return to the lab (approximately 1 week, 8 months, 16 months, and 24 months after Session 1) to complete a number of tasks. Data from the Actigraph and Actiwatch will be downloaded. Participants will have their height and weight measured and complete a packet of questionnaires. Participants will be scanned with the Hologic Discovery A dual energy X-ray absorptiometry (DXA) scanner. During the DXA scans, participants will wear a gown and asked to lie flat on a table. After their body is positioned by a technician, they will be still for 3 min in order for the scan to be completed. Female participants will take a urine pregnancy test. Those with a positive test will be excluded from the DXA scan and further study participation. During their final session (Session 5), participants will complete the DSM-5 Self-Rated Level 1 Cross-Cutting Symptom Measure – Adult [30], and if necessary, relevant DSM-5 Self-Rated Level 2 measures [31] will be completed. During their final session, participants will also complete an exit interview focused on such topics as motivation to join the study, easy and difficult aspects of the study, and whether they changed any of their health behaviors.

#### Compensation

Eligible participants will be provided $10 for completing Session 1 activities (screening/training session). Participants must complete at least five days of at-home data collection (sleep, activity, food/beverage, media and technology use) and attend Session 2 to receive $20. They must complete at least five days of at-home data collection and attend Session 3 to receive $30. Participants must complete at least five days of at-home data collection and attend Session 4 to receive $35. Finally, participants need to complete at least five days of at-home data collection and attend Session 5 in order to receive $45. In total, all eligible participants will receive $140 for providing data and participating in all study sessions. If they do not attend a session or provide five days of at-home data, they will not receive payment for the relevant time period. Participants who leave the university or are away from the university during Session 5 will receive an additional $25 for travel-related costs. Payments will be provided immediately after completing required tasks so that study withdrawal will be discouraged.

#### Training and mentorship program

The Parent announcement of the National Institutes of Health (NIH) R15 mechanism that funded this study requires a training and mentorship program. This study will use a tiered-mentorship program in which two PhD level faculty members (first and second authors) will directly supervise MS level graduate students, who will then directly supervise undergraduate students. The two faculty members will interact with the undergraduate students on occasion. Graduate students will serve as paid Project Coordinators and undergraduate students will be volunteer RAs. Potential RAs will be required to submit a vita, complete an application, and attend an in-person interview to determine if they are a good fit for the project. Manuals, demonstrations by Project Coordinators, roleplaying exercises between RAs, and observation of RAs will be used for training. RAs will be given constructive feedback as needed. Some RAs will be chosen to be Captains, allowing them to assign some laboratory tasks and monitor the work of other RAs. All RAs will be given the opportunity to attend professional development sessions provided by the Principal Investigator on topics such as creating a vita, preparing for graduate school, and career planning. Project Coordinators will attend a weekly meeting with faculty members and they will also receive daily guidance as needed via phone, e-mail, or in person.

### Measures

#### Screening

A screening interview developed for Project STARLIT will be used to assess for self-reported medication use and current or a history of a neurological problem, head injury, or sleep disorder. The DSM-5 Self-Rated Level 1 Cross-Cutting Symptom Measure – Adult [30] was used for two purposes: 1) to diagnose current mood, anxiety, and/or substance use disorders, and 2) to screen for current psychosis and current suicidal ideation or plans. The Level 1 measure is a 23-item self-report measure of 13 psychiatric domains. Participants rate how much or how often they have been bothered by each item on a 5-point Likert scale. A discussion will occur about any items rated as a two or higher (with the exception of substance use, psychosis, or suicidal ideation which were discussed when rated as one). Participants will then complete the relevant DSM-5 Self-Rated Level 2 measures if necessary [31]. The Level 1 and Level 2 measures will also be administered during Session 5.

#### Objective measurement of sleep and physical activity

Sleep patterns (i.e., sleep/wake cycles) will be assessed using the Phillips Respironics Actiwatch 2 Device. Minutes of physical activity (light-, moderate-, and vigorous-intensity) will be measured using the Actigraph wGT3x-BT accelerometer.

#### Measurement of food and beverages

Daily dietary intake data will be collected using the Automated Self-Administered 24-h (ASA24) Dietary Assessment Tool, version (2016), developed by the National Cancer Institute, Bethesda, MD. Participants first record all foods and drinks they consumed at each meal. Then, participants are asked for the specifics of each meal. Finally, participants review everything they entered and are asked if they have forgotten any food or drinks and are allowed to enter more if needed. The ASA24 provides detailed data on macronutrients and energy, vitamins, minerals, carotenoids, fats and cholesterol, specific fatty acids, and other substances.

#### Daily diary

The following information will be collected in a daily diary: sleep information (e.g., bedtime, sleep latency estimate, naps), substances used (caffeine, nicotine, alcohol, and drugs), media and technology used (time of day, duration, and the reason(s) for use of all electronic devices such as computers, smart phones, tables, televisions), prescribed and over-the-counter medications and herbal/nutritional supplements used (name, dosage, reason for use, and amount of time they had been taking the medication or supplement), and monitoring device removal (time of day, duration, and for what purpose it was removed).

#### Questionnaire packet

Participants will complete a packet of questionnaires during Sessions 2–5. The measures included in the packet are described next.

The Pittsburg Sleep Quality Index [PSQI [35]; is a 19-item self-report measure of sleep quality and disturbances over the past month. Each item is rated on a scale ranging from 0 to 3. This measure consists of seven “Component” scores: subjective sleep quality, sleep latency, sleep duration, habitual sleep efficiency, sleep disturbances, use of sleep medication, and daytime dysfunction. These seven scores can be summed to create an overall sleep quality score ranging from 0 to 21 with higher scores indicating poorer sleep quality. However, this measure is not used for providing clinical diagnoses.

The Epworth Sleepiness Scale [ESS [36]; is a self-report measure of excessive daytime sleepiness. Participants rate how likely they would be to fall asleep in eight different situations on a 4-point Likert scale. Responses are summed to create a sleepiness score ranging from 0 to 24, with higher scores indicating greater daytime sleepiness.

The Sleep Hygiene Practice Scale [SHPS [37]; is a 30-item self-report measure of daily activities and sleep habits that may impact sleep. This scale measures sleep hygiene practices on 4 domains: sleep schedule and timing, arousal-related behaviors, poor eating/drinking habits prior to sleep, and poor sleep environment. Participants rate how often they engage in specific behaviors on a 6-point Likert scale ranging from never to always. Higher scores indicate poorer sleep hygiene.

The Dysfunctional Beliefs and Attitudes about Sleep-16 [DBAS-16 [38]; is a 16-item self-report measure of sleep/insomnia related thoughts. Participants rate to what degree they agree or disagree with each statement on an 11-point Likert scale. A total score can be calculated by averaging the score of all 16 items, with a higher score being indicative of more dysfunctional beliefs and attitudes about sleep.

The Multidimensional Fatigue Inventory [MFI [39]; is a 20-item self-report measure of the five dimensions of fatigue: general, physical, and mental fatigue, reduced motivation, and reduced activity. Participants rate how much each statement applies to them on a 7-point Likert scale. After reverse scoring and summing of all items, higher scores are indicative of a higher degree of fatigue.

The trait version of the Food Cravings Questionnaire [FCQ-T [40]; is a 37-item self-report measure of trait cravings within individuals in various times and situations. This measure contains nine factors: 1) having intentions and plans to consume food, 2) anticipation of positive reinforcement that may result from eating, 3) anticipation of relief from negative states and feelings as a result of eating, 4) lack of control over eating, 5) thoughts or preoccupation with food, 6) craving as a physiological state, 7) emotions that may be experienced before or during food cravings or eating, 8) cues that may trigger food cravings, and 9) guilt from cravings and/or for giving into them. Participants rate the frequency of each item being true for them on a 6-point Likert scale. Items can be summed for a total score and for each factor, with higher scores indicating greater stable food cravings.

The Life Experiences Survey [LES [41]; is a 57-item self-report measure of events an individual may have experienced over the past year. There are two sections of the LES; the first is for all respondents and the second is only for students. Section 1 consists of 47 specific life events with three blank spaces at the end where participants can write in other events they have experienced. The second section consists of 10 items, which are experienced in an academic setting. For both sections, participants indicate the events they have experienced in the past year and specify whether the event occurred 0–6 months or 7 months-1 year prior. Also, participants report whether this event was positive or negative and how much the event impacted their life when it occurred. This is reported by a 7-point Likert scale ranging from − 3 to + 3. To score this measure, a positive change score is calculated by summing all of the events that were indicated as positive, and a negative change score is calculated by summing all of the events that were indicated as negative. A total change score is calculated by summing the positive change score and negative change score, which will represent the total amount of desirable and undesirable change that the participant experienced over the past year.

Participants will complete a self-assessment questionnaire of their physical development using pictures and explanatory text reflecting the Tanner stages [42]. Males are asked to evaluate their genital development and pubic hair growth. Females are asked to evaluate their breast development and pubic hair growth. Later stages indicate more advanced development.

Participants will complete a demographics questionnaire during Session 2. The demographics questionnaire was created by the researchers to collect typical demographic information such as sex, race, ethnicity, religion, current residence, and income. During Sessions 2 and 5, participants will complete a health questionnaire, which was created by the first author and has been used in multiple studies. This questionnaire contains a list of 47 medical conditions or diseases. Participants are asked to indicate which medical problems they have and describe how much the condition has impacted their life.

### Data analysis

The main objectives of Project STARLIT are to characterize trajectories of habitual sleep across the four time points, determine whether sleep trajectories are related to weight gain risk behaviors, and identify what factors may contribute to sleep trajectory group membership. In order to characterize the sleep trajectory groups, we will use finite mixture models adapted for the case where the analysis focuses on trajectories [43–49]. The conceptual underpinning of this model is that each participant is a member of one of an unknown number of latent classes with a unique “trajectory type”, and their class membership determines their mean trajectory. The primary analytic goals of that analysis are to determine both the number of trajectory groups, and their characteristics. To determine the number of classes, several plausible fixed numbers of groups will be examined and the number of trajectories that optimizes a given information-based model selection criterion will be used [46, 47, 49]; diagnostics based class separation (e.g., using relative entropy) will also be used to evaluate the model fit. While the hypothesized variable of interest is sleep duration, the data will be examined to determine if another variable may be a better representation of the pattern of data (e.g., mean difference between weekday and weekend sleep time, or the % of days under 6 h). If that is the case, separate analyses will be conducted for each variable of interest.

To determine whether sleep trajectories are related to weight gain risk behaviors, each individual will be assigned to a latent class (i.e., trajectory type) based on the maximum posterior probability, and the latent class label will be used as a categorical predictor in a series of ANOVA/regression models predicting the various weight gain risk outcomes (i.e., increased caloric intake, decreased physical activity, weight gain, and increased body fat composition). Lastly, using multinomial logistic regression models, the identification of what factors contribute to latent glass group membership will be examined. Specifically, the assigned latent class variable will be used as the outcome variable and baseline measures of sleep-risk as predictors (i.e., amount of media and technology usage, problematic sleep-related beliefs/behaviors).

### Sample size justification

The examination of sleep trajectories is a relatively novel approach and the power to detect differences among trajectory types is dependent on multiple factors [50]. Therefore, sample size is difficult to estimate prospectively with certainty for two reasons, 1) the size and separation of sleep trajectory types in our proposed sample is relatively unknown and 2) the size and separation of trajectory types is one of the most important factors for determining the power necessary to detect trajectory types. However, with a sample size of *N* = 200, trajectories with a medium and high level of heterogeneity (i.e, among slopes and/or intercepts) can be reliably detected; power > 80% and power > 95% [51]. Further, a brief simulation was conducted based on published sleep quality trajectories (albeit in a sample with different demographic characteristics) [52] and sleep quantity characteristics of young adults [i.e., setting a conservative with-in group standard deviation to 30 min [53];. With an estimated sample size of *N* = 300 (accounting for 20% attrition, *n* = 240) the model was able to recover the four sleep trajectories of previous research with a small level of variability.

### Data management plan

Both paper-and-pencil (e.g., substance use, media and technology use) and electronic (e.g., sleep, activity, food/beverage) data will be collected. Participants will have individual paper folders labeled with participant numbers and these data will be stored in locked cabinets located in the locked laboratory space. RAs will enter paper data into a database created by a Project Coordinator and different RAs will check data twice. Data from the monitoring devices will be downloaded on a computer in the laboratory. ASA24 data will be requested at the end of each data collection period. All electronic data will be backed-up on an external drive each week by a Project Coordinator. The external drive will be stored in a locked cabinet, located in the locked laboratory, when not in use. In order to minimize any missing data, which is an important issue given Project STARLIT is a longitudinal study, questionnaire packets will be checked immediately after completion while the participants are still present. Participants will be given reminders for scheduled appointments. RAs will use manuals and checklists to make sure all sessions have been conducted as planned and all data have been collected. All data will be cleaned and scored before data analysis begins.

## Discussion

Project STARLIT will examine whether there are differential patterns of habitual sleep (i.e., sleep trajectories) and determine if the patterns are related to weight gain risk over the first two years of college in a sample of college students recruited prior to beginning college. Additionally, Project STARLIT will identify what may contribute to the development of these sleep trajectories. There is existing research regarding whether short sleep is associated with risk for weight gain and obesity; however, there are limitations that will be addressed in the current study. Specifically, previous longitudinal investigations have relied heavily on self-report measures of sleep and energy expenditure/physical activity, which are low to moderately correlated with the associated objective measure outcomes [54, 55]. Therefore, objective measures and advanced techniques, such as DXA to assess body composition (i.e., fat mass and lean mass) will be used to more accurately assess the variables of interest. Improvements in measurement will be accomplished by using objective measures of habitual sleep, physical activity, and body fat composition.

In an effort to address limitations of previous sample characteristics (i.e., missed critical period in which habitual sleep changes have a maximum impact on weight gain), the recruitment of recent high school graduates, prior to entering college, will provide a unique opportunity. The observation and change in habitual sleep patterns (i.e., sleep trajectories) over an important developmental period have not been examined nor has there been an examination of sleep trajectories related to weight gain risk behaviors and outcomes.

Finally, Project STARLIT provides the opportunity to identify both intervention and prevention targets to be tailored to and tested with college students at a time when problematic unhealthy behaviors are potentially developing. If the current study hypotheses are supported, comprehensive and age-appropriate sleep interventions (e.g., addressing sleep hygiene, sleep scheduling, problematic beliefs) will be developed for implementation and evaluation during the first semester of college. Additional intervention targets may also be identified and would be included in the intervention to foster healthy eating, adequate physical activity, moderate sedentary leisure with or without technology usage, and emotional well-being. Therefore, Project STARLIT will likely suggest that an intervention to promote, teach, and maintain healthy behaviors is necessary.

## Abbreviations

ASA24: Automated Self-Administered 24-h Dietary Assessment Tool
BMI: body mass index
DBAS-16: Dysfunctional Beliefs and Attitudes about Sleep-16
DXA: dual-energy x-ray absorptiometry
ESS: Epworth Sleepiness Scale
FCQ-T: Food Cravings Questionnaire, Trait version
HSIRB: Human Subjects Institutional Review Board
LES: Life Experiences Survey
MFI: Multidimensional Fatigue Inventory
NIH: National Institutes of Health
PSQI: Pittsburg Sleep Quality Index
SHPS: Sleep Hygiene Practice Scale
